# Social prescribing link workers—A qualitative Australian perspective

**DOI:** 10.1111/hsc.14079

**Published:** 2022-10-22

**Authors:** Leah S. Sharman, Niamh McNamara, Shaun Hayes, Genevieve A. Dingle

**Affiliations:** ^1^ School of Psychology University of Queensland St Lucia Australia; ^2^ Nottingham Trent University Nottingham UK

**Keywords:** community participation, link workers, loneliness, social connection, social prescribing

## Abstract

Social prescribing (or community referral) is a model of healthcare designed to address social needs that contribute to poor health. At the heart of social prescribing programs is the link worker, who liaises between clients, health professionals and community organisations. Social prescribing is newly emerging in Australia but there are already calls for a large‐scale roll out. This research, therefore, aimed to understand Australian link workers' role and skills required, to determine where such a workforce could be drawn from in Australia, and to identify what training and resources are needed to support this potential new workforce. To explore these questions, interviews were conducted with 15 link workers in Queensland, New South Wales and Victoria, and the transcripts were analysed using thematic analysis. Participants were predominantly female (87%); and primarily had qualifications in social work (47%) or nursing (27%). Three overarching themes were identified: (1) skills of successful social prescribing, identifying that link work requires multifaceted social and emotional skills; (2) workforce issues, presenting that link workers experienced challenges such as a lack of available support and training, lack of public awareness of social prescribing and a lack of sustained funding; and (3) job fulfilment, related to link workers' sense of reward and accomplishment from the job. We suggest that fostering job fulfilment in conjunction with the provision of increased support, training and security will reduce feelings of overwork and burnout among link workers and likely lead to longevity in the role. Social prescribing has the potential to be hugely beneficial to clients and the community and fulfilling for link workers, provided that sufficient advocacy and resources are put in place.

**What is known about this topic?**

- A growing evidence base exists in relation to social prescribing link workers and the complexity of their roles.
- There is limited research on link workers experiences, particularly in relation to the resources required to continue their roles long term and the workforce capacity.
- There is also limited research on experiences of link workers outside of the United Kingdom where social prescribing is emerging.

**What this paper adds?**

- The present study is the first to investigate link workers' experiences in Australia and investigates how this workforce can be best utilised as social prescription grows in popularity.
- Results revealed that health workers are broadly trained in the skills needed to carry out successful link work, that there was a lack of support and training with high risks of burnout, and that despite these difficulties link workers felt a strong sense of reward and accomplishment in their work.
- This study makes recommendations on the types of support and training required to sustainably continue and expand social prescribing link work in Australia.

## 1 SOCIAL PRESCRIBING LINK WORKERS—A QUALITATIVE AUSTRALIAN PERSPECTIVE

It is estimated that 10% of general practice (GP) patients account for 30%–50% of appointments, yet these ‘frequent attenders’ often do not feel satisfied with the services they receive (Vedsted & Christensen, 2005). This is partly due to social needs that are not readily met in medical services, such as loneliness and social isolation (Cruwys et al., 2018). Patients whose health is impacted by social factors are typically prescribed medication and/or given referrals for psychotherapy and counselling (Maughan et al., 2016). However, these treatment options are not ideal. Despite loneliness being associated with an increased risk of depression (Erzen & Çikrikci, 2018), there is no evidence that antidepressants are effective for loneliness. Medications such as antidepressants nonetheless have high rates of over prescription in Australia (Wallis et al., 2021) with other referral avenues in psychology and counselling succumbing to critical short staffing and long wait times to access services (InPsych, 2021). A potential solution to these issues is social prescribing, a community‐based model of healthcare designed to address unmet social needs that contribute to poor health.

In social prescribing, clients are linked to a range of community services and social activities in order to address their social needs (GSPA, 2021; Kimberlee, 2013). Although there is no universal model (Kimberlee, 2013), social prescribing schemes commonly involve three components: (1) the individual is referred to the program, often by a GP or allied health professional; (2) the individual meets with a link worker (or ‘navigator’) to discuss their interests and needs and (3) the individual engages with a meaningful social activity within their local community (Dingle & Sharman, 2022; GSPA, 2021). Although many health providers offer a type of ‘light’ social prescribing such as signposting of available activities with possible follow‐up, high‐intensity or ‘holistic’ social prescribing aims to address clients' social determinants of health more comprehensively (Kimberlee, 2013).

In Australia, holistic models of social prescribing are emerging in primary care, aged care and community services despite no national or state level oversight (Aggar et al., 2021). Those based in primary care share many features in common with the United Kingdom, with one or more link workers based internally within a primary care service. Community programs are otherwise based in neighbourhood or community centres, with external GPs, allied health, or community workers able to identify socially isolated persons and refer them to the social prescribing scheme. These community‐based schemes, while focused on improving individual well‐being, also work towards building and enhancing the capacity of local communities (Morris et al., 2022). Although there are currently few such social prescribing programs in Australia the potential for growth is evident (CSSC, 2021), with strong interest from consumers, GPs and allied health professionals (RACGP & CHF, 2019). Further, the recent Queensland Parliamentary Inquiry into social isolation and loneliness recommended a large‐scale roll out of social prescribing in Queensland to help address these significant social issues (CSSC, 2021).

The potential for a new workforce of social health care professionals would be invaluable to reduce service loads and to provide more relevant and specific care for people feeling lonely and isolated. Indeed, research from service users and link workers themselves make clear that they are critical to the success of social prescribing (Kellezi, Wakefield, et al., 2019; Wildman, Moffatt, Penn, et al., 2019; Zurynski et al., 2020). However, questions arise about where a new and potentially large workforce might come from, and what skills, qualifications and accreditation are warranted, particularly given the lack of clarity for qualifications required in the United Kingdom (Bickerdike et al., 2017). The fast and widespread roll out of social prescribing in the United Kingdom following the Government's 2018 loneliness strategy (Department for Digital Culture Media and Sport, 2018), while ambitious, failed to appropriately coordinate resources to account the complexities of link work. This resulted in some criticisms of social prescribing on the grounds of a lack of role delineation for link workers, which were at times done by volunteers, and a lack of relevant training for new link workers (Bickerdike et al., 2017; Fixsen et al., 2020; Islam, 2020). Although the Queensland parliamentary inquiry report recommended this workforce be sourced from social workers, they may be drawn from other fields, and we do not know the type of workforce currently operating in Australia. Further, it remains to be seen what training and support may be required to upskill link workers to ensure an effective and safe workforce is developed for the future. Thus, the aims of this study are (i) to understand the experience of Australian link workers in relation to their role and skills required to identify where such a workforce could be drawn from and (ii) to understand link workers' experiences to determine what training and resources are needed to support this potential new social care workforce.

## 2 METHOD

### 2.1 Participants

Link workers at 10 social prescribing schemes found in Australia were approached to participate in the study. These 10 schemes were identified through chain referrals from other organisations and online searches. Due to a lack of link worker registration, the total number of link workers across these schemes could not be accurately determined. Most organisations employed one or two link workers, but some had over a dozen people doing link work as part of their job, such as nurse practitioners in some GP clinics. Overall, 15 link workers agreed to participate from eight social prescribing programs across Queensland, New South Wales and Victoria. Most participants (53%) were recruited from primary care‐based schemes, and the remaining 47% were recruited from community‐based schemes. All but two of the link workers were female (87%) and ages ranged from 24–61^1^ (*M* = 40.12). Participants had worked in a social prescribing role between 3 months and 7 years, with most having tertiary qualifications in social work (47%), nursing (27%), business (7%), community development (7%) and counselling (7%). Those without tertiary qualifications instead had external training in counselling skills. Link workers primarily worked with communities experiencing disadvantage, despite three programs being established in more affluent areas.

### 2.2 Ethics

Ethics approval for this research was granted by the University of Queensland Human Research Ethics Committee (2020001019). All participants provided written and/or verbal consent before the interviews. They were reimbursed with $60 shopping vouchers to compensate for their time.

### 2.3 Interviews and analysis

Semi‐structured interviews were conducted with questions focused on link workers' perspectives of the skills they use to link clients to groups, strategies to overcome and address client barriers, the positive aspects and drawbacks of their roles and their identity as a link worker. The interview guide (see Data S1) was designed through discussion with the research team in consultation with a link worker employed in both community and primary care settings. Interviews were conducted between June 2020 and November 2021. Across this period, link workers experienced different levels of COVID‐19 restrictions within each of their states and within each of their organisations. Interviews were conducted via phone (87%) or in person by a qualitative researcher (LS) and all interviews were recorded and transcribed verbatim. Interviews averaged 30 mins and ranged from 22 to 39mins.

Thematic analysis using an iterative process was employed to examine the data using a combination of an experiential approach and a constructionist approach (Terry et al., 2017). This was considered appropriate for the study because the inductive approach allowed participants to explore what was of meaning to them during the interviews, while the constructionist approach allowed the researchers to ask about aspects of link workers' experience guided by our research questions. The analysis followed a well‐established six‐phase procedure described by Braun et al. (2019) of (a) familiarity with data; (b) initial code generation; (c) construction of themes; (d) review of themes against the data; (e) further defining themes and (f) reporting results, with co‐authors commenting and providing feedback. Analysis of the data was led by LS with GD and SH reading responses and contributing to discussions of theme meanings and label refinement over three meetings, and UK‐based author (NM) providing ad hoc verbal and written input. The researchers have backgrounds in clinical, social and health psychology.

## 3 RESULTS

Link workers in the Australian context came from a broad range of professional backgrounds (see Participants) and it was clear that there was no strong sense of a link worker professional identity. Instead, link workers tended to identify as a member of their health or other professional role that was related to their qualification and training:

*My role title is a care coordinator. However, because I studied social work, I identify more in social work*. (LW2, primary care)

**FIGURE 1:**
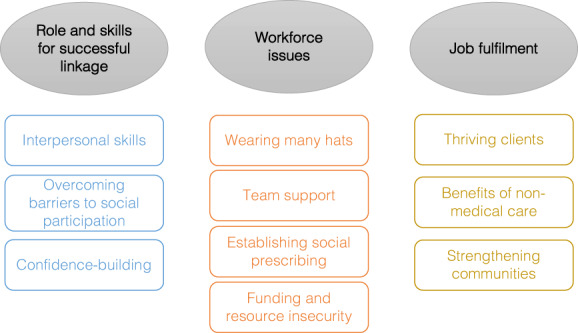
Thematic map

However, there were many common experiences of people doing link work. Three overarching themes were constructed from the data and are shown in the thematic map (Figure 1). These referred to the role and skills used by link workers that they saw as necessary for successful linkage of clients to community groups; the broader workforce experiences such as a lack of role clarity, lack of advocacy for social prescribing and a need for sustained funding and resources for social prescribing programs; and job fulfilment and satisfaction gained from seeing clients and communities thrive.

### 3.1 Role and skills for successful linkage

#### 3.1.1 Interpersonal skills

The skills needed to create a sense of trust and safety were commonly mentioned as fundamental for successful social prescribing. Rapport with clients allowed link workers to effectively communicate with clients and establish trust before pursuing new connections, and activities that might be outside their comfort zone:

*…the success of the program is really dependent on the relationship the wellbeing coordinator has with the client. If they can develop a relationship of trust and rapport, that they feel they can engage in something and be taken on that journey, then they feel safe to be able to do that…* (LW1, primary care)

Establishing a strong rapport sometimes resulted in clients viewing link workers as a friend or a counsellor, meaning that setting clear boundaries with clients was an important part of setting expectations in the relationship. Some link workers described this as a challenging process:

*So, I think, yeah, moving forward I have now a better picture and some more strategies to be able to put those boundaries and keep that scope of what a link worker is supposed to be and supposed to do*. (LW3, community)

Others recognised these skills, and actively trained their volunteers in boundary setting:

*…we've got a very big pool of volunteers, so doing some training with them around boundaries and that sort of thing as far as this is going to be tricky because you're actually going to be in the friend space, but you've got to be in the mentor space*. (LW6, community)

#### 3.1.2 Overcoming barriers to social participation

Link workers noted a high proportion of clients were vulnerable and experienced disadvantage, so elements such as costs, transport, accessibility and caregiving were highlighted as common barriers needing to be addressed. Supporting clients' agency in identifying and overcoming these barriers through a strength‐based approach was highly valued:

*If I recognise they have barriers, so transport, financial, mental health, then my priority [is] to work with them to overcome that*. (LW7, community)

Link workers often referred clients to other formal services for additional advocacy and support to access counsellors or psychologists, housing services, domestic violence, disability support and financial counselling. These would sometimes be based locally or within their own organisation. Further, community connections in these onward referrals allowed for better advocacy for a clients' needs and the execution of timely referrals:

*Social anxiety, depression, they play a huge part in those barriers to participation as well, and that's where coordinating care and linking in with other services is something that's really important for those people to be able to participate as well*. (LW3, community)

Clients often had multiple intersecting and layered complexities that, at times, made access to onward referrals more difficult. For example, cultural positioning, lack of access to translation services or physical safety concerns were each mentioned by link workers as compounding access barriers for clients:

*I think it can be a stressful job dealing with lots of barriers with not just the patient, but then with services as well, with access, and not just with social prescribing. But just in, often, every part of their life is complex*. (LW4, primary care)

#### 3.1.3 Confidence‐building

Preparing clients for joining their referred groups included ensuring clients felt emotionally safe and confident to attend and were motivated to continue attending. This included ensuring clients were well informed about the group and what it would be like to attend. This was done by sharing the link worker's experience of attending the group, or by attending the first group alongside them:

*We try and encourage them to take a support person, whether it's myself or our peer worker or a family member or carer to go to at least maybe the first one or two meetings*. (LW2 primary care)

It was also common to arrange an initial meeting with the group facilitator for clients to gauge their ‘fit’ for the group, to reduce social anxiety, increase feelings of belonging to the group and to check for the safe and equitable access to rooms and activities:

*Sometimes we have set up space for safe meetings with the facilitator as really informal meetings so that the client can have a look at the venue and get a sense of what it looks like when they get there*. (LW2 primary care)

Further, it involved discussions around clients' expectations of the group and how to prepare themselves if it did not go as planned, as well as follow‐up with clients after attendance to get feedback about the success of the group. This identified whether they needed to make any adjustments to client strategies while simultaneously getting a better understanding of the group for future referrals:

*So I always follow‐up. I always ask them for feedback about the activity, about the group, how they felt, were they made welcome, and all those sort of things*. (LW5 primary care)

### 3.2 Workforce issues

#### 3.2.1 Wearing many hats

Link workers described using diverse skills such as advocacy, counselling and crisis support as part of their clients' social prescribing journeys. The diversity of the role was further highlighted by their responsibilities outside of client contact, which not only required general community connection, but also involved advertising of the social prescribing program, and consistent updating of group activities (e.g. pandemic adjustments, group times, costs, etc.):

*But I think too, you're also part counsellor, you're a mental health support worker, you're an advocate for your client. So, you do wear many hats as a link worker*. (LW7, community)

This sense of wearing many hats and having to switch between roles was particularly true of those who had less support. Some link workers regarded this job diversity as a positive experience, providing variety and skill building:

*But because it's a small organisation and, luckily, I suppose, my skillset is quite broad, not necessarily expert at HR or marketing or any of that, but I'm okay at giving everything a go, which is lucky because I kind of have to do everything*. (LW15, community)

For others, these diverse tasks resulted in high burden and feelings of overwork:

*Overworked, yeah, yeah, or overwhelmed. And you just have to implement your self‐care strategies. Yeah, but I think long term, to be brutally honest, long term it probably isn't sustainable being just one*. (LW3, community)

#### 3.2.2 Team support

Multidisciplinary team support was viewed as desirable by most link workers, whether they were working in a team or not. Multidisciplinary teams included professionals such as GPs, social workers, peer support workers and even financial counsellors working alongside link workers. This strategy was highlighted as providing more substantial wrap‐around support for clients and allowing for simpler coordination and referral among link workers:

*I think for a program like this to make the biggest impact, it requires a coordinated approach, which means you need a team. You need a team of people that work together, but with specific roles so that you can provide that wraparound support for people*. (LW3, community)

Having teams involved in social prescribing was also considered as helpful for link workers to manage workloads and able to provide broad day to day support and problem solving in their roles:

*Although we were doing the same things, we still helped each other out. We still supported each other and you still had someone to bounce things off. And yeah, so that was manageable*. (LW3, community)

Several participants also felt that having small or no access to teams alongside a limited workforce of Australian link workers lead to feelings of isolation and lack of social support that was particularly true of community‐based link workers in this study:

*I think one of the drawbacks [is] I felt quite isolated myself in the work that I [am] trying to do*. (LW7, community)

#### 3.2.3 Establishing social prescribing

Link workers described few people in the Australian health, community, or public sectors knew about the social prescribing approach, what it entails, or what its benefits were. This dearth of information surrounding social prescribing meant that link workers have had to continuously explain and promote their services to stakeholders and potential clients. This was more keenly felt by those in the community sector where advertising was a particularly salient part of their role to encourage referrals within the community. For primary care‐based link workers this was less problematic as their direct referrers were already part of their service with knowledge of the scheme:

*I [do] lots of presentations at different organisations about three to four times a week, just to really get the word out. Because as we sort of said before, the link worker role and social prescribing … [isn't] as well known here in Australia*. (LW7, community)

Further, this lack of understanding by stakeholders resulted in link workers receiving inappropriate referrals into the program (e.g. people needing emergency relief, acute/specialised care or assistance completing applications for social or disability financial support):

*It's taken a long time probably to get people to get their head around what I do. Because they just see it as, “Oh, it's getting a lot of really inappropriate referrals”*. (LW6, community)

#### 3.2.4 Funding & resource availability

Link workers commonly experienced problems with accessing resources due to short‐term and insecure funding:

*So, we didn't have a lot funding. Literally, it paid for my salary and that was about it. So, I didn't have a lot of resources*. (LW7, community)

Further, funding insecurity was also apparent for other formal supports and third sector social groups that link workers referred into. In some cases, this resulted in limited staffing at group programs and was an obstacle to access quality resources for clients:

*… it's really difficult because a lot of services are funding‐based, [clients] are often part of the program short term and funding runs out*. (LW4, primary care)

### 3.3 Job fulfilment: Thriving clients and communities

#### 3.3.1 Thriving clients

Although link work was experienced as complex and difficult at times, link workers described it as satisfying and fulfilling. Positive client outcomes were impactful on all link workers interviewed who consistently described it as the most fulfilling aspect of link work.

*You get to empower people. You get to participate with them and walk alongside them*. (LW3 community)

These outcomes included seeing clients empowered, gain more confidence, make social connections and manage their health and social needs:

*I love seeing all of our members each week and seeing them grow and thrive and go off to do things*. (LW15 community)

#### 3.3.2 Benefits of non‐medical care

Increasing quality of life for clients outside of medical care was also seen by some as an advantage of social prescribing and something that ultimately added to their sense of satisfaction in their role. Some discussed how social prescribing targets the client's social engagement and sense of belonging to improve health‐related targets such as self‐efficacy, self‐confidence and self‐esteem. And others were more specific about providing them care that was more tailored to their social needs outside of the clinical space:

*… and passionate about seeing people given the opportunity and also probably not putting them in the clinical space when they don't necessarily need to be*. (LW6, community)

#### 3.3.3 Thriving communities

Fostering relationships with the community included assisting with the formation of new community and interest groups, connecting with existing activities and group facilitators and increasing referral pathways to include broader community services outside health care settings. The formation of these relationships made for easier access to groups and services, and fostered more supportive and accepting facilitators, which allowed clients to have a smoother transition to joining groups:

*But there's also, I spend a lot of time networking and getting to know groups and activities, as well as putting my feelers out for new referrers*. (LW3 community)

Outside individual client improvement, some link workers identified the access to social prescribing in local communities as strengthening bonds in the community. This went beyond increasing the number and size of groups in the community, instead focusing on improving relationships between individuals, families, social groups and organisations:

*And then from a community perspective, it's really just to see the community come together and want to really just reduce social isolation in our local community*. (LW7, community)

For some, there was a sense of support and championing from the broader community to tackle loneliness, where they saw it as a community issue, rather than an individual one:

*So we're putting it on for the [clients], but we all get benefits from being together and seeing people thrive and seeing people do stuff that they never imagined that they could do*. (LW15 community)

## 4 DISCUSSION

Social prescription has only recently begun in Australia with very few dedicated link workers across the country. However, the appetite for social prescribing programs is rising in this part of the world (CSSP, 2021; RACGP & CHF, 2019). This qualitative research aimed to understand Australian link workers' roles and skills to determine where such a workforce could be drawn from in Australia, and to identify what training and resources are needed to support this potential new workforce. Link workers described experiences that fell into three broad themes regarding the role and skills required for link work to be successful with clients, current issues arising from the workforce and sources of satisfaction and fulfilment from their role.

### 4.1 The link work skillset

Our results revealed that Australian link workers were primarily women who identified with their professions in health and social care, such as nursing and social work. However, the breadth of experience was diverse with link workers also having training in business, counselling (formal and informal) and community development. The experience and skills gained from these previous occupations were important for carrying out link work that involved interpersonal communication, planning and networking and responding to clients' needs through counselling and advocacy (Wildman, Moffatt, Penn, et al., 2019). The skills identified for the role were similar to those found in international studies with link workers and clients (Frostick & Bertotti, 2021; Holding et al., 2020; Wildman, Moffatt, Penn, et al., 2019; Wildman, Moffatt, Steer, et al., 2019) and further highlighted the integral role of link workers providing advocacy and safety for clients to successfully participate in social programs that are not simply accomplished through signposting (Stuart et al., 2022). Our results indicate that, generally, health professionals are well suited to the link worker role, given much of their prior training equips them with suitable and transferrable skills needed. Rather than restricting the link worker workforce to a single health profession such as social workers (CSSC, 2021), the flexibility of the social prescribing approach allows for this new workforce to be drawn from people with broader health training and skilled up to do important social health care.

To support this wider recruitment approach, access to training and guidelines in specific areas should be available for link workers to further their skills (Frostick & Bertotti, 2021). These could include interpersonal skills, managing client relationships, undertaking risk assessments, and broad understanding of the impacts of socio‐economic, environmental, health and emotional issues. Further, specific training in mental health first‐aid and trauma‐informed care principles would increase capability of working with and referring clients experiencing complex mental health‐related issues (Kimberg & Wheeler, 2019). Given the diversity of backgrounds able to successfully work in these roles, quality training and supervision would assist in identifying where further skill building is required and ensure that everyone has access to the knowledge and needs of the role (RACGP & CHF, 2019).

### 4.2 Social prescribing workforce

Responses from link workers regarding their funding security and resource availability prompted different responses dependent on the type of social prescribing model they were engaged in (community centres vs. primary care). Link workers within primary care typically had access to larger teams, supervision and a greater understanding from referrers about the program. Conversely, link workers set in community centres had smaller teams with fewer link workers and less understanding from their referrers about appropriate referrals for social prescribing. However, precarious funding was a problem across most programs. The different models of social prescribing exerted a big effect on how link work was done, namely, the degree of role diversity required and the burden of responsibility that was placed on link workers.

These results reiterate the difficulties of smaller and community‐driven models of social prescribing (Holding et al., 2020). Although there is a clear need for the community model to identify and assist socially isolated persons who do not regularly attend with a GP, further work is needed to increase the infrastructure and support available for those working in small teams. This includes sustained funding to ensure link workers are not working in silos by increasing funded positions to reduce workload and increase peer support and connection across sites. Not doing so will lead to increased burnout among link workers due to high workloads, which reliably predict emotional exhaustion and fatigue (Michielsen et al., 2004). Indeed, link workers indicated that the demand of their workloads was already causing feelings of exhaustion. Further, widespread education for consumers and referrers about what social prescribing is and its benefits would separately reduce the burden on link workers to educate others and the number of inappropriate referrals received (Islam, 2020). These modifications will also help partner organisations work together more effectively.

### 4.3 Community health and connection

Despite the challenges identified, link workers were proud of their programs and the successes they could achieve alongside their clients and communities. Although this sense of fulfilment was mainly focused on positive outcomes for individual clients, link workers also expressed satisfaction about their contribution to developing community connections. Through their outreach, link workers had increased awareness of loneliness in the community and helped to mobilise community members and groups to champion this issue.

Of course, while several studies have investigated the challenges of link work, to our knowledge, this is the first study to reveal circumstances of fulfilment and accomplishment in link work roles. Indeed, our results found that feelings of fulfilment were strong and widespread across all link workers interviewed. It is possible that engaging with and fostering elements of satisfaction in link work roles are likely to reduce burnout risks associated with a lack of meaning and reward (Cartwright & Holmes, 2006). Further, the association between overwork and low reward are predictors for emotional exhaustion and cynicism. Therefore, helping to identify and promote these sources of fulfilment and personal accomplishment within the linker worker role, alongside providing proactive support and training, will be key to reducing future burnout and turnover, and decrease interrupted care for clients.

Importantly, while there have been positive outcomes for communities thus far, a wider roll out of link worker programs could put resource pressure on the community and volunteer sectors currently helping to champion social prescribing as has been found in the United Kingdom (Bertotti et al., 2018; Holding et al., 2020; The Health Foundation, 2015; Tierney et al., 2020). It is therefore important that these sectors are adequately funded so increases in referrals do not lead to overburden and long wait times for access to group programs.

### 4.4 Research and policy implications

Social prescribing has the potential to increase the location and scope of health care beyond hospitals and health clinics to people's homes and communities (GSPA, 2021). For link workers, providing a service that could reduce time spent in clinical spaces brought an added sense of achievement. Social prescription, of course is able to provide greater access to health care targeting the social determinants of health and simultaneously reducing the pressure on limited hospital, GP and psychological services. Indeed, emerging evidence suggests that social approaches may be comparable in effectiveness to individualised care for issues such as depression, which is highly correlated with loneliness (Dingle et al., 2021). However, there remains a need for further controlled evaluations where the majority of social prescribing initiatives have been evaluated qualitatively (Bickerdike et al., 2017; Chatterjee et al., 2018; Ending Loneliness Together, 2021; Kellezi, Frings, et al., 2019). Strong quantitative long‐term evaluations are necessary to further identify what parts of social prescribing work best and why to further shape and improve social prescribing practices (Husk et al., 2020).

## 5 CONCLUSION

Aligned with the Australian Federal Government's consumer‐centred healthcare plan (CHF, 2018) and the Productivity Commission Mental Health reform (Productivity Commission, 2020), social prescribing has the potential to provide a ‘holistic health service’ where social, physical and mental health concerns can be addressed (Kellezi, Wakefield, et al., 2019). This study provides the first investigation into the experiences of social prescribing link workers in Australia and revealed both areas of positivity and areas in urgent need of sustained support and development. Given the strong evidence linking loneliness and social isolation with mental and physical health conditions and morbidity (Holt‐Lunstad & Steptoe, 2022), investment in social prescribing by all levels of Government is likely to produce cost savings for health and support thriving communities in Australia in the aftermath of the pandemic.

## AUTHOR CONTRIBUTIONS

All authors contributed to the conceptualisation and design of the study. LS analysed the data with input on themes from GD, NM and SH. All authors contributed to the interpretation of themes for the discussion. LS and GD contributed to the first draft writing. All authors reviewed and edited the manuscript. All authors have read and agreed to the final version of the manuscript.

## Supporting information


Data S1

## Data Availability

The data that support the findings of this study are available on request from the corresponding author. The data are not publicly available due to privacy restrictions.

## Endnote

^1^
One person chose to provide their age within a range and were excluded from the mean calculation.
