# Supplementary material for: Social prescribing link workers—A qualitative Australian perspective
Source: Health Soc Care Community. 2022 Oct 22;30(6):e6376–85. doi: 10.1111/hsc.14079 (PMC10092430; doi:10.1111/hsc.14079)
Supplement: Supplementary file 1 — Data S1 [file HSC-30-e6376-s001.docx]

Social Prescribers Experiences

1. Can you tell me about your regular role within your Social Prescribing Service?
2. From your experience to what extent do you believe social prescribing is a successful way to break social isolation/loneliness?
3. Could you describe some of the strategies you use to connect clients to groups?
4. What have been the common barriers for clients trying to engage with group programs through the social prescribing service?
5. When a client has been previously ‘unsuccessful’ in a group and decided not to continue, what other barriers, if any, do they experience when joining a new group?
6. In what ways have you managed to address and overcome these different barriers with your clients?
7. In what ways do the facilitators of community groups and programs help your clients to engage and feel like they belong?
8. Can you comment on the extent you identify as a link worker/wellbeing coordinator?
9. As a link worker/wellbeing co-ordinator, what do you feel are the positive aspects of your role?
10. Similarly, what do you feel are the drawbacks?
